# Worsening quality of life in asthmatics, non-obstructed smokers and ACO during the COVID-19 pandemic in patients from the ELSA-Brazil

**DOI:** 10.1016/j.clinsp.2025.100788

**Published:** 2025-09-24

**Authors:** Roselaine Maria Oliveira, Francine Maria de Almeida, Vitoria Caroline Queiroz, Anna Paula de Lima Costa, Renato Fraga Righetti, Edna Aparecida Leick, Paulo Lotufo, Isabela Bensenor, Itamar Souza Santos, Iolanda de Fatima Lopes Calvo Tibério

**Affiliations:** a Laboratório de Terapêutica Experimental-LIM20, Faculdade de Medicina, Universidade de São Paulo, São Paulo, SP, Brazil; b Departamento de Clínica Médica, Faculdade de Medicina, Universidade São Paulo, São Paulo, SP, Brazil; c Hospital Universitário da Universidade de São Paulo, São Paulo, SP, Brazil; d Hospital Sírio-Libanês, Serviço de Reabilitação, São Paulo, SP, Brazil

**Keywords:** Asthma, Pulmonary Disease, Chronic Obstructive, Asthma-Chronic Obstructive Pulmonary Disease Overlap Syndrome, Spirometry, Cytokines, COVID-19

## Abstract

**Introduction:**

Asthma-COPD Overlap (ACO) combines characteristics of both asthma and COPD, resulting in fixed airflow obstruction. During the COVID-19 pandemic, these patients may have experienced a decline in quality of life.

**Objectives:**

To identify demographic, biochemical, and inflammatory markers and their relationship with quality of life during the COVID-19 pandemic in individuals with ACO.

**Methods:**

This was a cross-sectional study including 174 participants from the ELSA-Brazil study in São Paulo during the COVID-19 pandemic. Participants were allocated into four groups: control (*n* = 42), asthma (*n* = 42), Non-Obstructed Smokers (NOS) (*n* = 38), and ACO (*n* = 49). Quality of life was assessed using the SF-36 short, CAT, and WHOQOL-bref questionnaires. Complete blood count and C-Reactive Protein (CRP) were analyzed, and serum levels of interleukins IL-4, IL-5, IL-6, IL-8, IL-13, IL-17, and TNF-α were measured using Luminex analysis. Pre- and post-bronchodilator performance of FVC, FEV1, FEV1/FVC ratio, and FEF 25 %‒75 % was evaluated. Correlation analyses were conducted between interleukins and questionnaires, blood count, and spirometry results.

**Results:**

Quality-of-life questionnaires showed poor mental, physical, and social health during the COVID-19 pandemic, with SF-36 indicating worsened functional capacity in the control and NOS groups, while CAT revealed a moderate impact in the ACO group. The asthma group had elevated IgE levels, and inflammation, as expressed by cytokines (TNF-α, IL-6, IL-8, IL-4, IL-17A, and IL-13), was highest in the NOS group. The ACO and asthma groups showed reduced post-bronchodilator values for FEV1, FEV1/FVC, and FEF 25 %‒75 %. Correlations were found between cytokines and quality of life, lymphocytes, leukocytes, neutrophils, and hemoglobin levels.

**Conclusion:**

During the pandemic, low quality of life levels were observed in all groups. IgE levels were higher in asthmatics, while the NOS group exhibited greater inflammation. Spirometry indicated lung function impairment in the asthma and ACO groups. Correlations were observed between inflammatory mediators, interleukins, and quality of life.

## HIGHLIGHTS

• Cytokines and lung function link to health decline worsened by COVID-19 and smoking.

## Introduction

Asthma affects between 1 % and 29 % of the global population,^1^ while Chronic Obstructive Pulmonary Disease (COPD) affects approximately 10 %.^2^ Asthma-COPD Overlap (ACO) is a more recently recognized condition, characterized by fixed airflow obstruction and clinical and inflammatory features of both diseases.^3^ Despite its clinical relevance, ACO still lacks a standardized definition due to limited scientific evidence.^3^

Diagnostic criteria typically include age over 40-years and fixed airflow obstruction, defined by a forced expiratory volume in one second to Forced Vital Capacity ratio (FEV1/FVC) of <0.7, a significant bronchodilator response (≥ 12 % and 400 mL increase in FEV1), history of smoking or biomass exposure, and the presence of asthma or atopy. Eosinophilia and elevated IgE levels are also frequently observed.^4^ Among smokers, lung function decline may occur even in the absence of spirometric obstruction, associated with inflammation.^5^^,^^6^

In January 2020, the World Health Organization (WHO) declared COVID-19 (Coronavirus Disease 2019) a global public health emergency.^7^ Prolonged symptoms such as fatigue, dyspnea, sleep disturbances, and reduced functional capacity were reported for months after infection, negatively impacting quality of life. However, the literature remains limited regarding the effects of COVID-19 in individuals with ACO or Non-Obstructed Smokers (NOS).

This study was conducted within the framework of the Brazilian Longitudinal Study of Adult Health (ELSA-Brazil), a multicenter initiative following public servants from six institutions.^8^ The objective was to identify demographic, biochemical, and inflammatory markers associated with ACO and the NOS group, and to examine their relationship with quality of life during the pandemic.

## Methods

The study was approved by the Ethics Committee of the University Hospital of the University of São Paulo under approval number 3.287.305. It was structured following the Strengthening the Reporting of Observational Studies in Epidemiology (STROBE) guidelines.^9^

### Study design

This is a field-based, exploratory study with a quantitative, observational, and cross-sectional approach. Regarding data type, the study includes both primary data (collected through analyses) and secondary data (obtained from the Longitudinal Study of Adult Health-ELSA-Brazil database). Sampling was intentionally selected based on participants' self-reported diagnoses.

### Setting

The participants included in this study were previously selected from the ELSA-Brazil cohort at the São Paulo investigation center^8^ following the third ELSA wave, which took place between 2017 and 2019.

ELSA-Brazil recruitment began in 2008 and involves continuous follow-up of its participants with periodic evaluations. The data generated have significantly influenced public policies within the Brazilian Unified Health System (SUS) by describing the prevalence and risk factors for cardiovascular diseases, diabetes, and dyslipidemia. Additionally, it assesses participants' awareness of their condition, as well as the treatment and control of these risk factors.^10^

The sample collection took place during the COVID-19 pandemic, from May to December 2022. Participants were invited via phone call and received an email and/or text message to confirm their attendance on the scheduled data collection day. They were instructed to fast for four hours and to avoid consuming alcoholic and/or caffeinated beverages within the 12 h preceding sample collection.

The collected samples were processed at the University Hospital, the Experimental Therapeutics Laboratory (LIM-20), affiliated with the Department of Clinical Medicine of the Faculty of Medicine of the University of São Paulo (FMUSP), and the Special Analysis Laboratory (Multi-user) LIM-03 of FMUSP.

### Participants

The authors included ELSA-Brazil participants who self-reported at study baseline as belonging to one of the following groups: (a) Control, (b) Asthma, (c) NOS, and (d) ACO.

Participants were excluded if they were unable to complete the self-administered questionnaire; had influenza or tested positive for COVID-19 at the time of data collection; did not agree to sign the Informed Consent Form (ICF); had spirometry results incompatible with a diagnosis of asthma, COPD, or other smoking-related obstructive conditions; or did not meet the criteria for inclusion in the control group.

### Variables

Medication use was considered a study variable, as it may influence spirometric, inflammatory, and quality-of-life-related findings.

Additionally, since participants undergo periodic medical examinations as part of their involvement in the ELSA-Brazil study, they are likely already aware of their health status and may adopt preventive care measures.

### Data sources / measurement

For all groups, data were collected on demographic parameters (gender, age, and medications used for respiratory disease treatment), quality-of-life assessments SF-36,^11^ WHOQOL-BREF,^12^ and CAT,^13^ and biochemical parameters (complete blood count for eosinophil and neutrophil counts, C-Reactive Protein [CRP], and Interleukin [IL] analysis, including IL-4, IL-5, IL-6, IL-8, IL-13, IL-17A, and Tumor Necrosis Factor [TNF-α]), measured using Magpix Milliplex (Luminex® Corp., Austin, Texas, USA).

Functional spirometric parameters were also evaluated, including FVC, FEV1 and the FEV1/FVC ratio.

### Quality of life and health status

#### Questionnaires

At the investigation center, in a calm and quiet environment, participants independently read and completed the following questionnaires, validated in Portuguese, on paper with a pen and without time constraints:

SF-36: Consists of 11 questions and 36-items covering eight components (domains or dimensions): functional capacity (10-items), physical aspects (4-items), pain (2-items), general health status (5-items), vitality (4-items), social aspects (2-items), emotional aspects (3-items), mental health (5-items), and one comparative question regarding current health perception compared to a year ago. Each domain generates a score ranging from 0 to 100, where 0 represents the worst score and 100 the best.^11^

WHOQOL-bref: Consists of 26 items, with the first two questions assessing self-perceived quality of life and satisfaction with health. The remaining 24 questions are divided into four domains: physical, psychological, social relationships, and environment.

In the WHOQOL-bref, only one question is used: the one with the highest correlation with the total score, calculated as the average of all responses. It includes five response scales for evaluation (each item is rated on a scale from 1 to 5, where 1 represents the worst condition and 5 the best): “very poor to very good” (evaluation scale), “very dissatisfied to very satisfied” (evaluation scale), “not at all to extremely” (intensity scale), “none to complete” (capacity scale), and “never to always” (frequency scale).

The average score in each domain reflects an individual's perception of satisfaction with various aspects of life and their overall quality of life. Higher scores indicate better perception.^12^

CAT (COPD Assessment Test): Evaluates the health status of COPD patients by quantifying the impact of common symptoms of the disease (cough, sputum production, chest tightness, shortness of breath when climbing hills or stairs, limitations in daily activities, confidence in leaving home, sleep, and energy levels).

The question scores range from 0 to 5, with a total CAT score ranging from 0 to 40; higher scores indicate worse health status. A cutoff score of ≥10 suggests a compromised health condition. The impact of COPD symptoms on patients' lives can be classified into four categories based on the CAT score: low (1–10), moderate (11–20), high (21–30), and very high (31–40).^13^

### Analysis of inflammatory mediators’ expression in blood

The internal laboratory performed a complete blood count and PCR measurement. Of the 15 mL of blood collected, 2 mL were centrifuged at 800 rpm at 20 °C, with a G-force of approximately 143.23 for 5-minutes, and the serum was frozen and stored at −80 °C until the analysis of IL-4, IL-5, IL-6, IL-8, IL-13, IL-17, and Tumor Necrosis Factor (TNF-α) was conducted.

The analysis of interleukins was performed in duplicates to minimize the risk of error at the Laboratory of Special Analyses (Multiuser) LIM03 of FMUSP. The authors used the xMAP technology from Luminex®, which employs color-coded fluorescent beads that covalently bind to capture antibodies. These capture antibodies are directly targeted against the desired biomarker. After a series of washes to remove unbound proteins, detection antibodies are added to form the “sandwich” complex, followed by the addition of streptavidin conjugated with phycoerythrin.

For standard reconstruction and curve construction, the bead dilutions, samples, antibodies, streptavidin-phycoerythrin, and wash solution followed the instructions provided in the kit manual, according to the manufacturer's guidelines.

In brief, color-coded beads coated with capture antibodies against the biomarkers of interest were pipetted into a 96-well plate. Subsequently, samples from patients, controls, the standard curve, and blanks were added and incubated on a plate shaker (IKA MTS 2/4 digital) according to the manufacturer's instructions. After washing using a magnetic washer (Bio-plex PRO II Wash Station), detection antibodies were added to the samples.

The beads were retained on the plate by a magnet during the washing process. Next, streptavidin-phycoerythrin was added, which emits a fluorescent signal when excited by a Light-Emitting Diode (LED) from the Magpix Milliplex bead reader (Luminex Corp, Austin, Texas, USA), using the Xponent 4.2 software (Luminex Corp, Austin, Texas, USA). The data analysis was then conducted using Milliplex Analyst 5.1 software (EMD Millipore).

The biomarker concentrations were determined based on the standard curve adjusted to the average Fluorescence Intensity (MFI) in relation to pg/mL. The biomarker levels were expressed in pg/mL according to the standard curve obtained in the assay.

### Spirometry

Participants were directed to a room in the outpatient clinic for spirometry testing, accompanied by the evaluator. The test was performed while the participants were seated in a chair, following the evaluator's instructions for each phase of the procedure.

Spirometry evaluates lung function ‒ pre- and post-bronchodilator ‒ and adhered to the criteria proposed by the American Thoracic Society in 1995,^14^ using the portable Koko® device (Pds Instrumentation Inc., Louisville, USA). The references used were based on the consensus established by the Brazilian Society of Pulmonology and Phthisiology (SBPT), as outlined by Pereira et al.^15^

The following parameters were assessed: FVC; FEV1; Forced Expiratory Flow (FEF 25 %‒75 %); and the ratio between FEV1/FVC.

For the test, disposable mouthpieces were used, and the parameters were measured 15-minutes before and after the administration of 400 mg of Salbutamol Sulfate®.

### Bias

The study population consisted of employees and former employees from a single university, which limits the sample’s representativeness in relation to the general population and, consequently, the generalizability of the findings. Additionally, the clinical stability of the participants, the absence of pre-pandemic data, and the small sample size may have influenced the results obtained. The cross-sectional study design also represents a limitation, as it prevents causal interpretations.

Initially, the study protocol included sputum analysis and Fractional Exhaled Nitric Oxide (FeNO) measurement. However, due to the risk of contamination among the team and participants during the COVID-19 pandemic, these analyses were excluded to reduce the risk of infections during the data collection and sample handling period.

### Sample size

Sample size was determined based on the number of participants who self-reported having or not having a respiratory disease diagnosis in the ELSA-Brazil database. The ELSA-Brazil database from the University of São Paulo contains 4752 participants (which includes: 2266 controls; 1309 ex-smokers; 665 current smokers; 237 asthmatic individuals; 121 asthmatic ex-smokers; 67 asthmatic current smokers; 18 COPD patients; 19 COPD ex-smokers; 12 COPD current smokers; 10 individuals with both asthma and COPD; 21 individuals with both asthma and COPD ex-smokers; 7 individuals with both asthma and COPD current smokers).

Groups were allocated according to the self-reported diagnosis from participants during the ELSA-Brazil study, and diagnoses were confirmed through spirometry.

It was planned to obtain 40 participants per group, and a total of 174 participants were enrolled in the study. Data from this population (self-reported respiratory disease) were extracted from the database, which included the following groups: ASTHMA group (individuals with asthma, according to GINA 2024),^1^ Non-Obstructed Smoker group (NOS ‒ individuals self-reporting COPD, according to GOLD 2024),^2^ however, they do not yet have the pulmonary obstruction characteristic of COPD on spirometry, Therefore, NOS does not mean that they are healthy, but rather that the disease has not yet manifested in a typical way in pulmonary function tests. ACO group (asthmatic smokers or ex-smokers, according to GINA-GOLD 2017),^3^ and control group (individuals without a history or prior complaints of diagnosed chronic respiratory diseases).

Randomization and blinding procedures were not applied due to the design characteristics and the observational nature of the study.

### Quantitative variables

The quantitative variables in this study were analyzed on a continuous scale, enabling the comparison of different data at a single point in time, such as age and the values obtained from various tests and analyses. To enhance the interpretation and analysis of the results, the data were categorized based on the confirmed diagnosis from spirometry. This categorization facilitated the formation of groups according to the results obtained, ensuring a more structured and comparable analysis.

### Statistical methods

The Shapiro-Wilk normality test was performed for all analyses. For results following a parametric distribution, a One-Way Analysis of Variance (ANOVA) was used for multiple comparisons, with results expressed as mean and standard deviation. For non-parametric results, a One-Way Analysis of Variance by Ranks (Kruskal-Wallis) was applied, with results expressed as median and percentiles.

Correlations were assessed using Spearman’s test between each cytokine (TNF-α, IL-6, IL-8, IL-4, IL-17A, IL-13) and the following parameters: hemoglobin, hematocrit, leukocytes, neutrophils, lymphocytes, eosinophils, IgE, PCR, spirometric results (FVC, FEV1, FEV1/FVC, FEF 25 %–75 %), and SF-36 domains with low scores (Functional Capacity, Pain, General Health Status, Vitality, Social Aspects, and Mental Health). Additionally, correlations were made with all WHOQOL-bref domains, showing reduced values in Physical, Psychological, Social Relationship, Environment, and General Perception, as well as with the CAT. The authors used the Interquartile Range (IQR) to represent data dispersion and Cohen's d to indicate the effect size between two groups.

The analysis was conducted using the Sigma® 11.0 statistical package for Windows, with a significance level of *p* ≤ 0.05 adopted for all analyses.

Multiple correlations were tested, and given the exploratory nature of the study, the authors prioritized identifying potential relationships that could generate hypotheses for future investigations, especially when the results are consistent with existing literature. Regarding data presentation, the authors adopted a statistical description based on the distribution of the variables, which facilitates understanding and interpretation of the results. Detailed raw data can be made available upon request for greater transparency.

## Results

A total of 174 participants from the ELSA-Brazil cohort were evaluated, with three participants excluded for not meeting the criteria for any group after spirometry analysis.

### Stages of the data collection and analysis process



ELSA-Brazil, Brazilian Longitudinal Study of Adult Health; USP, University of São Paulo; ICF, Informed Consent Form; CRP, C-Reactive Protein; mL, Milliliter; NOS, Non-Obstructed Smoker; ACO, Asthma-COPD overlap; FMUSP, Faculty of Medicine of the University of São Paulo.

A total of 42 asthmatic participants (ASTHMA group), consisting of 22 men and 20 women; 38 individuals who self-reported having COPD but lacked confirmatory spirometry (NOS group), consisting of 19 men and 19 women; 49 active or former smoking asthmatics (ACO group), consisting of 17 men and 32 women; and 42 controls ‒ participants without comorbidities (18 men and 24 women) ‒ were included in the study.

The mean age of these participants was 63 years (ranging from 48 to 87 years), comprising 95 women and 76 men, all residents of São Paulo. All participants in the control and ASTHMA groups were non-smokers, while the NOS group had a higher proportion of smokers and ex-smokers compared to the ACO group.

The use of medications such as beclomethasone, betamethasone, budesonide, ketotifen, ciclesonide, disodium cromoglycate, desloratadine, dexamethasone, fenoterol, fluticasone, formoterol, betamethasone phosphate, hydrocortisone, ipratropium, methylprednisolone, methylprednisone, mometasone, montelukast, prednisolone, prednisone, salbutamol, salmeterol, theophylline, and xylometazoline was higher in the ACO group compared to the other groups.

There was also a decrease in post-bronchodilator FEV1/FVC in Liters (L) and ( %) among participants in the ASTHMA group (*L* = 0.76 [0.66–0.81], % = 98.00 [87.00–102.25]) and ACO group (*L* = 0.74 [0.66–0.80], % = 93.50 [84.50–102.50]) compared to the control group (*L* = 0.82 [0.77–0.85], % = 104.00 [99.00–107.00]) (*p* < 0.05). Additionally, Forced Expiratory Flow between 25 % and 75 % of FVC (FEF 25 %–75 %) post-bronchodilator was lower in Liters (L) and ( %) in the ASTHMA group (*L* = 2.01 [1.38–2.75], % = 75.00 [58.25–115.50]) and ACO group (*L* = 1.65 [1.12–2.44], % = 70.00 [46.00–107.50]) compared to the control group (*L* = 2.60 [2.05–3.72], % = 114.00 [92.25–130.25]) and NOS group (*L* = 2.46 [1.97–3.08]), with only the control group showing a % of 114.00 [92.25–130.25] (*p* < 0.05) (Table 1).

**Table 1:** Pulmonary function test after bronchodilator use.

		**Spirometric Results**			
		**Control**	**ASTHMA**	**NOS**	**ACO**
FVC	**Post (L)**	**3.25 (2.87‒3.87)**	**3.13 (2.80‒3.44)**	**3.16 (2.75‒4.06)**	**2.70 (2.32‒3.47)**
	IQR	1.00	0.64	1.31	1.15
	*C*ohen’s *d*	‒	0.19	0.10	0.69
	**Post (%)**	**93.15 (11.72‒1.85)**	**88.70 (15.04‒2.35)**	**93.24 (12.96‒2.13)**	**86.12 (14.21‒2.05)**
	IQR	13.57	17.39	15.09	16.26
	Cohen’s *d*	‒	0.39	0.008	0.63
VEF1	**Post (L)**	**2.68 (0.79‒0.12)**	**2.29 (0.80‒0.12)**	**2.67 (0.70‒0.11)**	**2.10 (0.71‒0.10)**^a^^,^^b^
	IQR	0.91	0.92	0.81	0.81
	Cohen’s *d*	‒	0.58	0.02	0.91
	**Post (%)**	**98.00 (86.00‒104.75)**	**84.00 (66.75‒97.25)**^a^	**92.00 (84.00‒100.25)**	**83.50 (63.50‒91.50)**^a^^,^^b^
	IQR	18.75	30.50	16.25	28.00
	Cohen’s *d*	‒	0.75	0.46	0.82
VEF1/FVC	**Post (L)**	**0.82 (0.77‒0.85)**	**0.76 (0.66‒0.81)**^a^	**0.79 (0.76‒0.82)**	**0.74 (0.66‒0.80)**^a^
	IQR	0.08	0.15	0.06	0.14
	Cohen’s *d*	‒	0.58	0.27	0.73
	**Post (%)**	**104.00 (99.00‒107.00)**	**98.00 (87.00‒102.25)**^a^	**100.00 (97.00‒103.00)**	**93.50 (84.50‒102.50)**^a^
	IQR	8.00	15.25	6.00	18.00
	Cohen’s *d*	‒	0.70	0.77	1.09
FEF 25 %‒75 %	**Post (L)**	**2.60 (2.05‒3.72)**	**2.01 (1.38‒2.75)**^a^	**2.46 (1.97‒3.08)**	**1.65 (1.12‒2.44)**^a^^,^^b^
	IQR	1.67	1.37	1.11	1.32
	Cohen’s *d*	‒	0.79	0.09	1.01
	**Post (%)**	**114.00 (92.25‒130.25)**	**75.00 (58.25‒115.50)**^a^	**97.00 (83.00‒105.75)**	**70.00 (46.00‒107.50)**^a^
	IQR	38.00	57.25	22.75	61.50
	Cohen’s *d*	-	1.01	0.55	1.25

VC, Forced Vital Capacity; FEV1, Forced Expiratory Volume in the first second; FEV1/FVC, Forced Expiratory Volume in the first second over Forced Vital Capacity (Tiffeneau index); FEF 25 %‒75 %, Forced Expiratory Flow at 25 %‒75 % of FVC. Pulmonary function test performed before and after bronchodilator (Salbutamol®), presented in liters and percentages ( %); Percentage variation post-bronchodilator. Non-parametric data are presented as median (25 %‒75 %) and parametric data as mean ± standard deviation. Quantitative variables were compared using the ANOVA test with the SigmaStat 11.0 statistical software.

a vs. Control groups.

b vs. NOS group. *p* ≤ 0.05 was considered statistically significant. IQR, Interquartile Range, represents the spread of the data between the first quartile (Q1) and the third quartile (Q3). Higher values indicate greater variability. **Cohen’s *d* interpretation:** 0.2 = small | 0.5 = medium | 0.8 = large (control vs. ASTHMA, NOS and ACO).

The WHOQOL-bref quality of life questionnaire showed no significant differences among the groups across all evaluated domains. However, in the SF-36 questionnaire, the functional capacity domain had lower values in the control and NOS groups compared to the ACO group (*p* < 0.05). Additionally, CAT scores were lower in the control and ASTHMA groups compared to the ACO group (*p* < 0.05) (Table 2).

**Table 2:** Quality of life assessment.

	**Results of quality-of-life questionnaires**			
	**Control**	**ASTHMA**	**NOS**	**ACO**
**SF-36**				
Capacity Functional	**48.70 (48.70‒48.60)**^a^	**48.70 (48.60‒48.90)**	**48.65 (48.60‒48.80)**^a^	**48.90 (48.70‒49.10)**
IQR	0.10	0.30	0.20	0.40
Cohen’s *d*	‒	0	0.43	−0.93
Physical Limitation	**98.00 (98.00‒98.30)**	**98.00 (98.00‒98.50)**	**98.00 (98.00‒98.30)**	**98.00 (98.00‒98.80)**
IQR	0.30	0.50	0.30	0.80
Cohen’s *d*	‒	0	0	0
Pain	**19.15 (19.00‒19.30)**	**19.10 (19.00‒19.30)**	**19.10 (19.00‒19.40)**	**19.30 (19.07‒19.40)**
IQR	0.30	0.30	0.40	0.33
Cohen’s *d*	‒	0.23	0.19	−0.65
General Condition of Health	**24.00 (23.90‒24.10)**	**24.00 (23.90‒24.20)**	**24.00 (23.90‒24.20**	**24.10 (24.00‒24.20)**
IQR	0.20	0.30	0.30	0.20
Cohen’s *d*	‒	0	0	−0.68
Vitality	**19.10 (19.10‒19.30)**	**19.10 (19.00‒19.20)**	**19.10 (19.00‒19.20)**	**19.20 (19.00‒19.30)**
IQR	0.20	0.20	0.20	0.30
Cohen’s *d*	‒	0	0	−0.53
Social Aspects	**23.90 (23.80‒24.10)**	**23.90 (23.80‒24.10)**	**23.90 (23.80‒24.10)**	**23.90 (23.80‒24.00)**
IQR	0.30	0.30	0.30	0.20
Cohen’s *d*	‒	0	0	0
Aspects Emotional	**98.00 (98.00‒98.30)**	**98.00 (98.00‒98.00)**	**98.00 (98.00‒98.00)**	**98.00 (98.00‒98.30)**
IQR	0.30	0.00	0.00	0.30
Cohen’s *d*	‒	0	0	0
Mental Health	**19.00 (19.00‒19.10)**	**19.00 (18.90‒19.10)**	**19.00 (18.90‒19.10)**	**19.00 (18.90‒19.10)**
IQR	0.10	0.20	0.20	0.20
*C*ohen’s *d*	‒	0	0	0
WHOQOL-bref				
Physical	**16.00 (14.30‒17.70)**	**16.30 (13.70‒17.70)**	**16.00 (14.90‒17.70)**	**15.40 (13.70‒16.60)**
IQR	3.40	4.00	2.80	2.90
Cohen’s *d*	‒	−0.11	0	0.26
Psychological	**15.30 (14.70‒16.70)**	**16.00 (15.30‒17.60)**	**16.00 (14.70‒18.00)**	**16.00 (15.15‒17.30)**
IQR	2.00	2.30	3.30	2.15
Cohen’s *d*	‒	−0.44	−0.35	−0.45
Social Relationship	**16.00 (14.70‒16.00)**	**16.00 (13.30‒16.00)**	**16.00 (13.30‒17.30)**	**14.70 (13.30‒16.00)**
IQR	1.30	2.70	4.00	2.70
Cohen’s *d*	‒	0	0	0.83
Environment	**15.00 13.50‒16.00)**	**15.50 (13.70‒17.00)**	**15.25 (13.50‒17.00)**	**15.00 (13.50‒16.00)**
IQR	2.50	3.30	3.50	2.50
Cohen’s *d*	‒	0.23	0.11	0
General Perception of Health	**16.00 (16.00‒18.00)**	**16.00 (16.00‒18.00)**	**16.00 (14.00‒18.00)**	**16.00 (15.50‒16.00)**
IQR	2.00	2.00	4.00	0.50
Cohen’s *d*	‒	0	0	0
Total	**15.50 (14.50‒16.90)**	**15.80 (14.50‒16.80)**	**15.45 (14.20‒16.90)**	**15.10 (14.20‒16.30)**
IQR	2.40	2.30	2.70	2.10
Cohen’s *d*	‒	0.17	−0.03	−0.24
CAT				
CAT – Total	**7.00 (3.00‒12.00)**^a^	**7.50 (3.00‒13.00)**^a^	**8.00 (6.00‒13.00)**	**12.00 (5.75‒16.00)**
IQR	9.00	10.00	7.00	10.25
Cohen’s *d*	‒	0.07	0.17	0.70

SF-36, Medical Outcomes Study 36-Item Short-Form Health Survey (0 = worst health status, 100 = best health status); WHOQOL-bref, The World Health Organization Quality of Life Assessment (0 % = worst quality, 100 % = best quality of life); CAT, COPD Assessment Test (COPD impact: 6–10 = mild; 11–20 = moderate; 21–30 = severe; 31–40 = very severe). Non-parametric data are presented as median (25th–75th percentile). Quantitative variables were compared using ANOVA in the SigmaStat 11.0 statistical program. ^a^ vs. ACO groups. *p* ≤ 0.05 was considered significant. IQR, Interquartile Range, represents the spread of the data between the first Quartile (Q1) and the third Quartile (Q3). Higher values indicate greater variability. **Cohen’s *d* interpretation**: 0.2 = small | 0.5 = medium | 0.8 = large (control vs. ASTHMA, NOS and ACO).

No significant differences were found among the groups regarding hemoglobin (g/mL of total blood), hematocrit ( %), leukocytes, neutrophils (mm³), lymphocytes (mm^3^ and %), or eosinophils (mm^3^ and %).

Regarding immunoglobulin IgE levels (IU/mL), elevated levels were observed in the ASTHMA group [78.0 IU/mL (42.0–144.0)] compared to the control [37.5 IU/mL (8.1–63.0)] and NOS groups [21.0 IU/mL (12.0–50.0)] (*p* < 0.05 for all comparisons).

C-reactive protein (CRP, mg/L) levels were analyzed in the control [1.3 mg/L (0.7–4.5)], ASTHMA [1.8 mg/L (0.9–4.3)], NOS [1.0 mg/L (0.5–2.5)], and ACO [2.1 mg/L (1.0–2.8)] groups, with no significant differences observed among them.

Serum TNF-α levels were elevated in the NOS group compared to the control, ASTHMA, and ACO groups (*p* < 0.05). IL-6 levels were higher in the NOS group than in the ASTHMA group (*p* < 0.05). IL-8 levels were greater in the NOS group compared to the control and ACO groups (*p* < 0.05). IL-4 levels were elevated in the NOS group compared to the ASTHMA and ACO groups (*p* < 0.05). IL-17A levels were higher in the NOS group than in the control and ASTHMA groups (*p* < 0.05). IL-13 levels were increased in the NOS group compared to the ASTHMA and ACO groups (*p* < 0.05). However, IL-5 levels did not differ among the groups (Table 3).

**Table 3:** Serum cytokines.

	**Cytokine Result**			
**Cytokines, Pg/mL**	**Control**	**ASTHMA**	**NOS**	**ACO**
TNF-α	**2.43 (1.76 – 3.12)**^a^	**2.43 (1.76 – 3.12)**^a^	**3.12 (2.69 – 3.83)**	**2.43 (1.76 – 3.12)**^a^
IQR	1.36	1.36	1.14	1.36
Cohen’s *d*	‒	0	0.74	0
IL-6	**1.79 (1.39 – 2.20)**	**1.39 (1.39 – 2.20)**^a^	**2.20 (1.79 – 2.62)**	**1.79 (1.39 – 2.20)**
IQR	0.81	0.81	0.83	0.81
Cohen’s *d*	‒	−0.67	0.67	0
IL-8	**3.31 (2.61 – 4.25)**^a^	**3.46 (2.61 – 4.78)**	**4.67 (3.62 – 6.68)**	**3.62 (2.21 – 4.85)**^a^
IQR	1.64	2.17	3.06	2.64
Cohen’s *d*	‒	0.10	0.74	0.19
IL-4	**7.91 (6.59 – 9.24)**	**6.59 (4.09 – 9.24)**^a^	**12.02 (6.59 – 12.02)**	**6.59 (6.59 – 9.24)**^a^
IQR	2.65	5.15	5.43	2.65
Cohen’s *d*	‒	−0.44	1.29	−0.67
IL-17 A	**7.92 (7.92 – 9.06)**^a^	**7.92 (7.92 ‒ 9.06)**^a^	**9.06 (7.92 – 9.06)**	**7.92 (7.92 – 9.06)**
IQR	1.14	1.14	1.14	1.14
Cohen’s *d*	‒	0	1.36	0
IL-13	**303.74 (244.03 – 303.74)**	**244.03 (213.37 – 332.94)**^a^	**332.94 (274.13 – 361.75)**	**244.03 (213.37 – 332.94)**^a^
IQR	59.71	119.57	87.62	119.57
Cohen’s *d*	‒	−0.85	0.53	0.85
IL-5	**4.32 (4.74 – 4.89)**	**4.32 (3.74 – 4.89)**	**4.89 (4.32 – 4.89)**	**4.32 (3.74 – 4.89)**
IQR	0.15	1.15	0.57	1.15
*C*ohen’s *d*	‒	0	1.84	0

Pg/mL, Picograms per Milliliter; TNF-α, Tumor Necrosis Factor-alpha; IL, Interleukin. Non-parametric data are presented as median (25 %–75 %). Quantitative variables were compared using ANOVA in the SigmaStat 11.0 statistical program.

Significance: *p* ≤ 0.05. NOS group indicated by ^a^. IQR, Interquartile range, represents the spread of the data between the first quartile (Q1) and the third Quartile (Q3). Higher values indicate greater variability. **Cohen’s d interpretation**: 0.2 = small | 0.5 = medium | 0.8 = large (control vs. ASTHMA, NOS and ACO).

The authors found weak to very weak negative correlations between general health status and TNF-α, IL-6, IL-8, IL-4, IL-17A, IL-13, and IL-5 (*p* < 0.05). Functional capacity showed a very weak negative correlation with IL-4 and IL-13 (*p* < 0.05). Social aspects exhibited a very weak negative correlation with IL-17A, trending toward statistical significance.

**Fig. 1:**
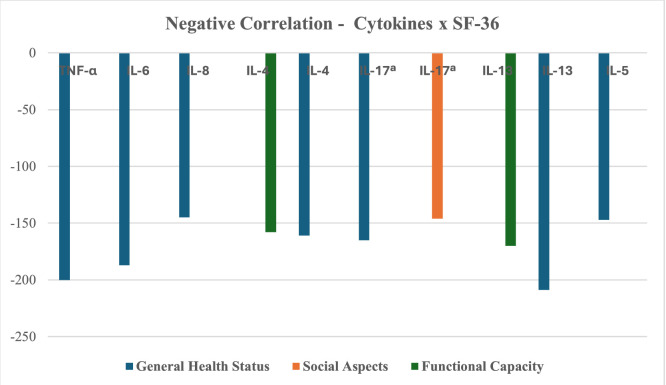
Correlation between cytokines × SF-36. Negative correlation between cytokines × SF-36 attributes.

**Fig. 2:**
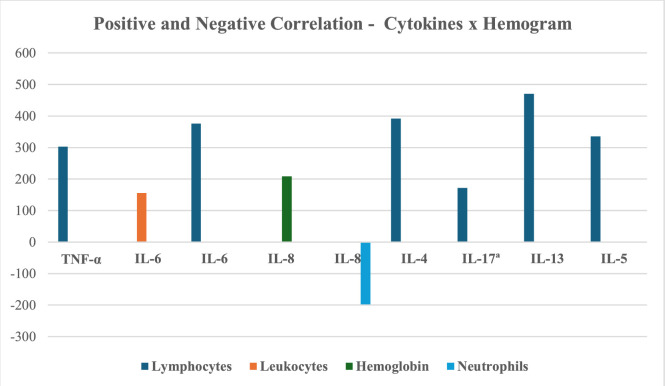
Correlation between cytokines × hemogram. Positive and negative correlation between cytokines × blood count components.

Lymphocytes displayed positive correlations, ranging from very weak to moderate, with TNF-α, IL-6, IL-4, IL-17A, IL-13, and IL-5 (*p* < 0.05). Leukocytes showed a very weak positive correlation with IL-6 (*p* < 0.05). Hemoglobin exhibited a weak positive correlation with IL-8, while neutrophils showed a very weak negative correlation with IL-8 (*p* < 0.05). Only significant correlations are reported (Table 4, Fig. 1, Fig. 2).

**Table 4:** Spearman correlation.

		**Results of Correlations**		
**Cytokines × Attributes**		**Correlation Coefficient**	**p-value**	**Result**
SF-36				
TNF-α	General Health Status	−0.200	0.0120	Negative correlation, weak, with significant p.
IL-6	General Health Status	−0.187	0.0194	Negative correlation, very weak, with significant p.
IL-8	General Health Status	−0.145	0.0703	Negative correlation, very weak, with a tendency towards significance.
IL-4	Functional Capacity	−0.158	0.0476	Negative correlation, very weak, with significant p.
IL-4	General Health Status	−0.161	0.0445	Negative correlation, very weak, with significant p.
IL-17ª	General Health Status	−0.165	0.0394	Negative correlation, very weak, with significant p.
IL-17ª	Social Aspects	−0.146	0.0684	Negative correlation, weak, with significant trend.
IL-13	Functional Capacity	−0.170	−0.170	Negative correlation, weak, without significant p.
IL-13	General Health Status	−0.209	0.00861	Negative correlation, weak, with significant p.
IL-5	General Health Status	−0.147	0.0671	Negative correlation, very weak, with trend to p significant.
Hemogram				
TNF-α	Lymphocytes	0.303	0.303	Positive correlation, weak, with a tendency towards significant p.
IL-6	Leukocytes	0.156	0.0520	Positive correlation, very weak, with significant p.
IL-6	Lymphocytes	0.376	0.00000153	Positive correlation, weak, with significant p.
IL-8	Hemoglobin	0.208	0.00910	Positive correlation, weak, with significant p.
IL-8	Neutrophils	−0.198	0.0133	Negative correlation, very weak, with significant p.
IL-4	Lymphocytes	0.392	0.000000511	Positive correlation, weak, with significant p.
IL-17ª	Lymphocytes	0.172	0.0314	Positive correlation, very weak, with significant p.
IL-13	Lymphocytes	0.470	0.000000000776	Positive correlation, moderate, with significant p.
IL-5	Lymphocytes	0.335	0.0000211	Positive correlation, weak, with significant p.

Quantitative variables were compared using Spearman's correlation test in the SigmaStat 11.0 statistical software. A p-value ≤0.05 was considered statistically significant. For correlation interpretation, the following classification was used: 0.00 to 0.19 = very weak correlation; 0.20 to 0.39 = weak correlation; 0.40 to 0.69 = moderate correlation; 0.70 to 0.89 = strong correlation; 0.90 to 1.00 = very strong correlation (Ciências do Desporto, 2024). Positive coefficients indicate that as the value of one variable increases, the value of the other also tends to increase. Negative coefficients indicate that as one variable increases, the other tends to decrease (Frost, 2021).

### Summary of results



COVID-19, Coronavírus Disease 2019; ACO, Asthma-COPD overlap; IgE, Immunoglobulin E; TNF-α, Tumor Necrosis Factor-alpha; IL, Interleukin; NOS, Non-Obstructed Smoker; SF-36, Short Form 36 Health Survey.

## Discussion

The present study revealed compromised quality of life across all groups. Spirometry demonstrated worse lung function in the ASTHMA and ACO groups, with elevated IgE levels in ASTHMA and greater inflammation in the NOS group, highlighting the impact of smoking even in the absence of pulmonary obstruction. Additionally, a correlation was observed between inflammatory cytokines and quality of life, reinforcing the role of inflammation in its deterioration.

This study was conducted with a cross-sectional design, without prior data for longitudinal analysis. However, its findings can serve as a basis for future longitudinal studies aiming to verify and expand on the observed results. Therefore, the conclusions and correlations presented refer solely to this single point of evaluation.

The SF-36 questionnaire showed reduced functional capacity in the control and NOS groups compared to the ACO group, with all groups presenting scores below 50 in several domains. According to Brazilian normative data for the Short Form-36, version 2, the closer the score is to zero, the worse the quality of life.^11^

The WHOQOL-bref questionnaire showed no significant differences between groups, aligning with Kobayashi et al.,^16^ who indicated that ACO does not have a worse prognosis than stable COPD. However, Lázár et al.^17^ suggest that quality of life worsens in patients with exacerbations, influenced by smoking and comorbidities. Scores below 18 % indicate a significant impact on quality of life, associated with functional limitations, persistent symptoms, emotional distress, and social and environmental restrictions.^12^

The ACO group showed a moderate impact on the CAT, higher than the control and ASTHMA groups, differing from Karloh et al.,^13^ who found higher scores in COPD patients.

The study, conducted at the end of the COVID-19 pandemic, showed low SF-36 and WHOQOL-bref scores across all groups, indicating declines in mental, physical, and social health. However, the absence of pre-pandemic data, confounding factors such as limited access to healthcare services, and lockdown measures make it difficult to attribute these changes solely to COVID-19. In the United States, there was an increase in psychiatric diagnoses and levels of anxiety and depression following infection with the virus.^18^ The presence of SARS-CoV-2 in the amygdala and hippocampus, areas related to mood and emotions, has been associated with symptoms such as insomnia, depression, and anxiety.^19^ These factors may have contributed to the participants' decline in quality of life, which could be further investigated in future studies. Among the 174 participants, 49 had COVID-19, but all were vaccinated.

The spirometric findings confirm the association between smoking and progressive lung function loss described by Ritchie et al.^5^ In the NOS group, systemic inflammation was observed even without spirometric confirmation of COPD, while the ACO group responded to bronchodilators, suggesting mild ACO, similar to mild asthma. Participants' increased attention to treatment during the pandemic underscores the importance of future follow-ups to monitor the progression of respiratory diseases and the impact of COVID-19 on quality of life.

Additionally, elevated IgE levels were found in the ASTHMA group, consistent with the findings of Yue,^20^ who associated high IgE with asthma, particularly the allergic form, which is characterized by early onset, atopic history, and elevated IgE levels.^21^

The study by Leung et al.^22^ reports elevated IgE levels in individuals with ACO and a history of asthma, which aligns with these findings, where IgE levels were highest in the ASTHMA group, followed by the ACO group. Additionally, greater systemic inflammation was observed in the NOS group in the cytokine analyses (TNF-α, IL-6, IL-8, IL-4, IL-17A, and IL-13), possibly due to the lack of specific treatment in this group.

Song et al.^23^ identified a significant association between tobacco-induced epigenetic aging and increased levels of the pro-inflammatory cytokines IL-8 and IL-6, which are linked to a higher risk of lung cancer and other respiratory diseases. Although Daloee et al.^24^ did not observe this relationship directly in his study, he cites other research reporting significantly elevated levels of TNF-α and IL-6 in aged rats chronically exposed to cigarette smoke, supporting the present findings. Similarly, Silva et al.^12^ described increased IL-6 expression in mice exposed to cigarette smoke and its role in the development of emphysema.

Gutiérrez-Romero et al.^6^ highlight that cigarette smoke and airborne particles induce the production of IL-6, IL-8, and TNF-α. This is consistent with the present findings of elevated cytokine levels in the NOS group. Additionally, smokers without chronic obstructive bronchitis exhibit plasma cells that express IL-4 and IL-5, with IL-4 promoting mucus secretion and exacerbating lung inflammation. In this study, IL-4 levels were elevated in the NOS group, corroborating the findings of Gutiérrez-Romero et al.,^6^ while IL-5 did not show significant differences among the four analyzed groups.

Exposure to cigarette smoke induces IL-17A expression in the airway epithelium, which amplifies inflammation and mucus production, acting in an autocrine manner and playing a key role in the early stages of COPD.^25^ In the present study, the NOS group showed elevated IL-17A levels despite the absence of typical spirometric features of COPD.

Elevated IL-13 levels in the NOS group, despite the absence of spirometric obstruction, indicate systemic inflammation associated with the asthmatic phenotype.^26^ This highlights the need for early monitoring and anti-inflammatory interventions to prevent progression to COPD, along with long-term follow-up to assess the predictive potential of IL-13 in the development of ACO.

In this study, Spearman's correlation analysis revealed negative associations between overall health status and various cytokines (TNF-α, IL-6, IL-8, IL-4, IL-17A, IL-13, IL-5), as well as negative correlations between functional capacity and IL-4/IL-13, and between social aspects and IL-17A. Conversely, a positive correlation was observed between lymphocytes and the cytokines TNF-α, IL-6, IL-4, IL-17A, and IL-13, as well as between leukocytes and IL-6, and between hemoglobin and IL-8, while neutrophils exhibited a negative correlation with IL-8. These findings align with existing literature, according to Schoormans et al.,^27^ various biological pathways influence quality of life, suggesting that immunological treatments could contribute to improving patient well-being.

This exploratory study, conducted during the COVID-19 pandemic, analyzed the asthma, ACO, and NOS groups. Although the NOS group did not show changes in lung function, it presented the highest levels of inflammation and the poorest quality of life. The clinical stability of participants and the absence of pre-pandemic data may have influenced the results. Pandemic-related restrictions also limited tests such as FeNO and sputum collection. Despite the small sample size, the correlations identified, although of low magnitude, are consistent with the literature and highlight the need for larger studies to confirm these findings. The cross-sectional design limits causal interpretations, such as the relationship between cytokine levels and quality of life.

Another limitation of the study is selection bias, as participants were employees and former employees of a single institution, which reduces the sample's representativeness. Additionally, the inclusion criteria limited the group's diversity. Future studies should expand diagnostic methods and include a broader range of biomarkers to improve the accuracy of the findings.

Despite these limitations, spirometry revealed a reduced bronchodilator response in the asthma and ACO groups, with declines in FEV1, FEV1/FVC, and FEF 25 %–75 %, indicating lung impairment. The SF-36 questionnaire identified worse functional capacity in the control and NOS groups, while the CAT indicated a moderate impact on quality of life in the ACO group. The ASTHMA group exhibited elevated IgE levels, while systemic inflammation, marked by increased levels of TNF-α, IL-6, IL-8, IL-4, IL-17A, and IL-13, was highest in the NOS group. Mild correlations were observed between these cytokines and various markers, including SF-36 scores, lymphocytes, leukocytes, neutrophils, and hemoglobin. Additionally, participants reported a decline in mental, physical, and social health during the COVID-19 pandemic.

Clinically, the findings indicate that patients with asthma and ACO have lung impairment and reduced bronchodilator response, requiring close monitoring. The elevated systemic inflammation in the NOS group suggests the need for early monitoring to prevent disease progression. The impact on quality of life and decline in mental and physical health during the pandemic highlight the importance of an integrated clinical approach, including psychosocial aspects.

This study was funded by FAPESP (2018/02,537–5, 2019/26,449–0, and 2022/02,510–5), LIM20-HC-FMUSP, and CNPq (01 060,115.00 SP).

## Availability of data and materials

The datasets used and/or analyzed during the current study are available from the corresponding author on reasonable request.

## Ethics statement

The studies involving human participants were reviewed and approved by the Ethics Committee of the Faculdade de Medicina da Universidade de São Paulo, number 3.287.305. The patients/participants provided this written informed consent to participate in this study.

## Conclusion

All participants experienced a decline in quality of life during the COVID-19 pandemic. Spirometry revealed lung impairment in the ASTHMA and ACO groups, while the NOS group exhibited increased systemic inflammation. Elevated IgE levels were observed exclusively in the ASTHMA group. Additionally, correlations were identified between inflammatory mediators, cytokines, and quality of life.

## Authors’ contributions

Roselaine Maria Oliveira: Conceptualization; investigation; data curation; formal analysis; writing-original draft; supervision.

Francine Maria de Almeida: Data curation; formal analysis; writing-review & editing.

Vitoria Caroline Queiroz: Investigation; data curation; formal analysis.

Anna Paula de Lima Costa: Investigation; data curation.

Renato Fraga Righetti: Writing-review & editing.

Edna Aparecida Leick: Resources; project administration.

Paulo Lotufo: Resources; project administration.

Isabela Bensenor: Resources; supervision.

Itamar Souza Santos: Data curation; resources.

Iolanda de Fátima Lopes Calvo Tibério: Conceptualization; supervision; writing-review & editing.

## Funding

The authors thank the Fundação de Amparo à Pesquisa do Estado de São Paulo FAPESP (n° 2018/02,537–5).

## Declaration of competing interest

The authors declare no conflicts of interest.

## Appendix Supplementary materials

Image, application 1
