# Supplementary material for: Worsening quality of life in asthmatics, non-obstructed smokers and ACO during the COVID-19 pandemic in patients from the ELSA-Brazil
Source: Clinics (Sao Paulo). 2025 Sep 24;80:100788. doi: 10.1016/j.clinsp.2025.100788 (PMC12495339; doi:10.1016/j.clinsp.2025.100788)
Supplement: Supplementary file 1 [file mmc1.docx]

Dear reviewer, I would like to add that, if the submission is accepted, we are committed to making all data used in our analyses publicly available through a public repository hosted on an artificial intelligence platform. This will ensure transparency and public access to the collected information.
